# 2,3,6-Trichloro-5-(trichloro­meth­yl)pyridine

**DOI:** 10.1107/S1600536812035404

**Published:** 2012-08-15

**Authors:** Xue-mei Zhu, Li-jun Pei, Zhao-sheng Cai, Zhan-qian Song, Shi-bin Shang

**Affiliations:** a College of Chemical and Biological Engineering, Yancheng Institute of Technology, Yinbing Road No. 9 Yancheng, Yancheng 224051, People’s Republic of China; b Institute of Chemical Industry of Forest Products, Chinese Academy of Forestry, Key and Open Laboratory on Forest Chemical Engineering, SFA, Nanjing 210042, Jiangsu Province, People’s Republic of China

## Abstract

The title compound, C_6_HCl_6_N, lies on a mirror plane, the asymmetric unit conataining a half-mol­ecule. Weak intra­molecular C—H⋯Cl contacts are observed.

## Related literature

For biological background, see: Okorley & Dietsche (1988 ▶). For the synthetic procedure, see: Allphin *et al.* (1993 ▶); For a related structure, see: Fun *et al.* (2011 ▶).
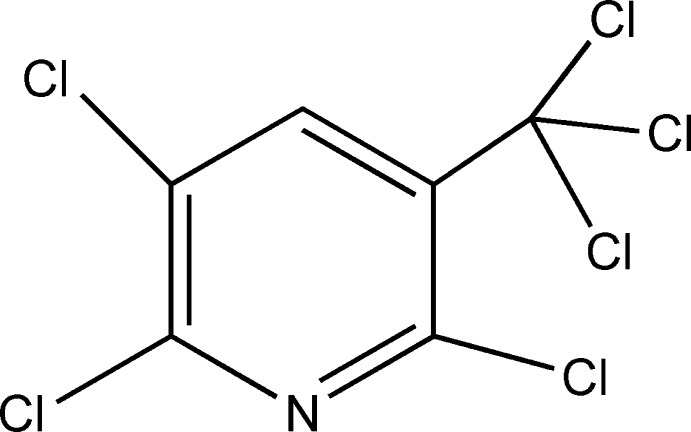


## Experimental

### 

#### Crystal data

C_6_HCl_6_N
*M* *_r_* = 299.78
Orthorhombic, 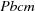

*a* = 8.3100 (17) Å
*b* = 17.018 (3) Å
*c* = 7.3160 (15) Å
*V* = 1034.6 (4) Å^3^
*Z* = 4
Mo *K*α radiation
μ = 1.61 mm^−1^
*T* = 293 K
0.30 × 0.20 × 0.20 mm

#### Data collection

Enraf–Nonius CAD-4 diffractometer
Absorption correction: ψ scan (North *et al.*, 1968 ▶) *T*_min_ = 0.644, *T*_max_ = 0.739
1985 measured reflections
1033 independent reflections
779 reflections with *I* > 2σ(*I*)
*R*_int_ = 0.063
3 standard reflections every 200 reflections intensity decay: 1%

#### Refinement

*R*[*F* ^2^ > 2σ(*F* ^2^)] = 0.040
*wR*(*F* ^2^) = 0.123
*S* = 1.01
1033 reflections
77 parameters
H-atom parameters constrained
Δρ_max_ = 0.26 e Å^−3^
Δρ_min_ = −0.39 e Å^−3^

### 

Data collection: *CAD-4 Software* (Enraf–Nonius, 1985 ▶); cell refinement: *CAD-4 Software*; data reduction: *XCAD4* (Harms & Wocadlo, 1995 ▶); program(s) used to solve structure: *SHELXS97* (Sheldrick, 2008 ▶); program(s) used to refine structure: *SHELXL97* (Sheldrick, 2008 ▶); molecular graphics: *SHELXTL* (Sheldrick, 2008 ▶); software used to prepare material for publication: *SHELXTL*.

## Supplementary Material

Crystal structure: contains datablock(s) I, global. DOI: 10.1107/S1600536812035404/pv2577sup1.cif

Structure factors: contains datablock(s) I. DOI: 10.1107/S1600536812035404/pv2577Isup2.hkl

Supplementary material file. DOI: 10.1107/S1600536812035404/pv2577Isup3.cml

Additional supplementary materials:  crystallographic information; 3D view; checkCIF report

## Figures and Tables

**Table 1:** Hydrogen-bond geometry (Å, °)

*D*—H⋯*A*	*D*—H	H⋯*A*	*D*⋯*A*	*D*—H⋯*A*
C1—H1*A*⋯Cl5	0.93	2.48	2.944 (5)	111

**Fig. 1.**
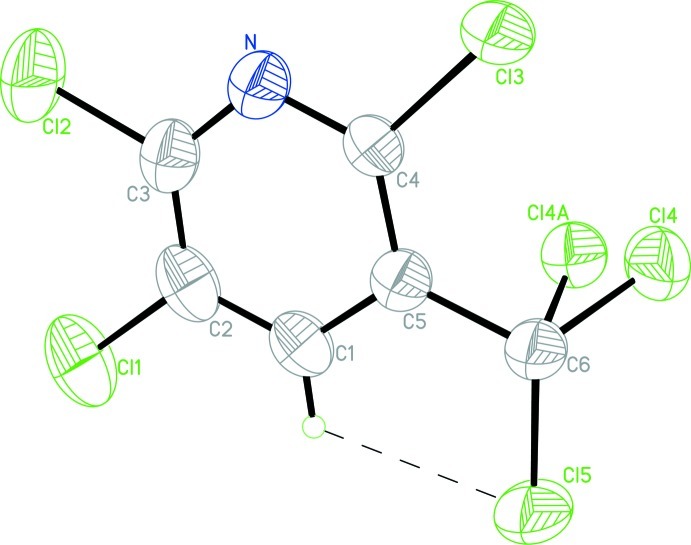
The molecular structure of the title compound with the atom numbering scheme. Displacement ellipsoids are drawn at the 50% probability level. H atom ise presented as small spheres of arbitrary radius. [Symmetry code A: x, y, 1/2-z]

**Fig. 2.**
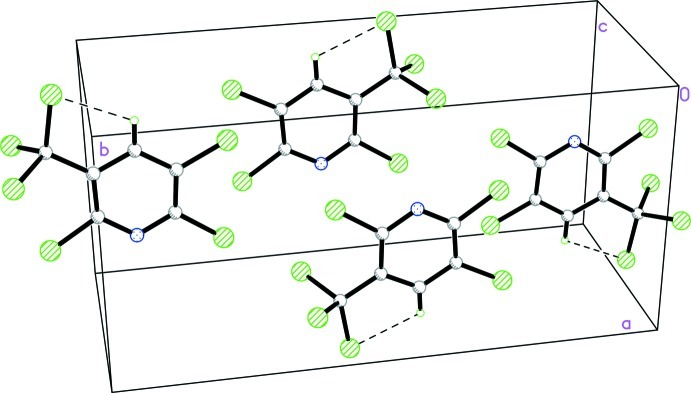
A packing diagram for the title compound showing C—H···Cl intra-molecular hydrogen bonds (dashed lines).

## supplementary crystallographic information

### Comment

Polychloropyridines derivatives are useful as intermediates for production of biological compounds (Okorley & Dietsche, (1988). Herein, we report the crystal structure of the title compound (Fig. 1). The bond distances and angles in the title compound agree very well with the corresponding bond distances and angles reported in a closely related compound (Fun *et al.*, 2011).

### Experimental

The title compound was synthesized by the chlorination of 2-chloro-5-chloromethyl pyridine using chlorine gas as a chlorinating agent in the presence of ultraviolet radiation and in the presence of WCl_6_ for 6.0 h by following a reported synthetic procedure (Allphin *et al.*, 1993). The crystals of the title compound were obtained from a solution of 1,2-dichloroethane by evaporating the solvent slowly at room temperature in about 5 d.

### Refinement

The only H atom was positioned geometrically and constrained to ride on C1 with C—H = 0.93 Å and *U*_iso_(H) = 1.2*U*_eq_(C).

### Crystal data

**Table:**

C_6_HCl_6_N	*F*(000) = 584
*M**_r_* = 299.78	*D*_x_ = 1.925 Mg m^−^^3^
Orthorhombic, *P**b**c**m*	Mo *K*α radiation, λ = 0.71073 Å
Hall symbol: -P 2c 2b	Cell parameters from 25 reflections
*a* = 8.3100 (17) Å	θ = 10–13°
*b* = 17.018 (3) Å	µ = 1.61 mm^−^^1^
*c* = 7.3160 (15) Å	*T* = 293 K
*V* = 1034.6 (4) Å^3^	Block, colorless
*Z* = 4	0.30 × 0.20 × 0.20 mm

### Data collection

**Table:**

Enraf–Nonius CAD-4 diffractometer	779 reflections with *I* > 2σ(*I*)
Radiation source: fine-focus sealed tube	*R*_int_ = 0.063
Graphite monochromator	θ_max_ = 25.4°, θ_min_ = 2.4°
ω/2θ scans	*h* = −10→10
Absorption correction: ψ scan (North *et al.*, 1968)	*k* = −20→0
*T*_min_ = 0.644, *T*_max_ = 0.739	*l* = −8→0
1985 measured reflections	3 standard reflections every 200 reflections
1033 independent reflections	intensity decay: 1%

### Refinement

**Table:**

Refinement on *F*^2^	Secondary atom site location: difference Fourier map
Least-squares matrix: full	Hydrogen site location: inferred from neighbouring sites
*R*[*F*^2^ > 2σ(*F*^2^)] = 0.040	H-atom parameters constrained
*wR*(*F*^2^) = 0.123	*w* = 1/[σ^2^(*F*_o_^2^) + (0.078*P*)^2^] where *P* = (*F*_o_^2^ + 2*F*_c_^2^)/3
*S* = 1.01	(Δ/σ)_max_ = 0.001
1033 reflections	Δρ_max_ = 0.26 e Å^−^^3^
77 parameters	Δρ_min_ = −0.39 e Å^−^^3^
0 restraints	Extinction correction: *SHELXL97* (Sheldrick, 2008), Fc^*^=kFc[1+0.001xFc^2^λ^3^/sin(2θ)]^-1/4^
Primary atom site location: structure-invariant direct methods	Extinction coefficient: 0.077 (6)

### Special details

**Table:**

Geometry. All e.s.d.'s (except the e.s.d. in the dihedral angle between two l.s. planes) are estimated using the full covariance matrix. The cell e.s.d.'s are taken into account individually in the estimation of e.s.d.'s in distances, angles and torsion angles; correlations between e.s.d.'s in cell parameters are only used when they are defined by crystal symmetry. An approximate (isotropic) treatment of cell e.s.d.'s is used for estimating e.s.d.'s involving l.s. planes.
Refinement. Refinement of *F*^2^ against ALL reflections. The weighted *R*-factor *wR* and goodness of fit *S* are based on *F*^2^, conventional *R*-factors *R* are based on *F*, with *F* set to zero for negative *F*^2^. The threshold expression of *F*^2^ > σ(*F*^2^) is used only for calculating *R*-factors(gt) *etc*. and is not relevant to the choice of reflections for refinement. *R*-factors based on *F*^2^ are statistically about twice as large as those based on *F*, and *R*- factors based on ALL data will be even larger.

### Fractional atomic coordinates and isotropic or equivalent isotropic displacement parameters (Å^2^)

**Table:**

	*x*	*y*	*z*	*U*_iso_*/*U*_eq_	
N	0.5025 (5)	0.4324 (2)	0.2500	0.0467 (9)	
Cl1	0.8417 (2)	0.26884 (8)	0.2500	0.0806 (6)	
C1	0.8339 (6)	0.4268 (3)	0.2500	0.0478 (11)	
H1A	0.9457	0.4241	0.2500	0.057*	
Cl2	0.45826 (19)	0.28195 (8)	0.2500	0.0753 (5)	
C2	0.7445 (7)	0.3584 (3)	0.2500	0.0537 (12)	
Cl3	0.47402 (13)	0.58193 (7)	0.2500	0.0569 (4)	
C3	0.5783 (6)	0.3642 (3)	0.2500	0.0486 (11)	
Cl4	0.81252 (10)	0.63147 (5)	0.05227 (15)	0.0619 (4)	
C4	0.5902 (5)	0.4969 (2)	0.2500	0.0399 (10)	
Cl5	1.06686 (13)	0.55710 (8)	0.2500	0.0672 (5)	
C5	0.7586 (5)	0.4991 (2)	0.2500	0.0418 (10)	
C6	0.8554 (5)	0.5748 (2)	0.2500	0.0451 (11)	

### Atomic displacement parameters (Å^2^)

**Table:**

	*U*^11^	*U*^22^	*U*^33^	*U*^12^	*U*^13^	*U*^23^
N	0.0472 (18)	0.042 (2)	0.051 (2)	−0.0058 (15)	0.000	0.000
Cl1	0.1128 (13)	0.0423 (7)	0.0867 (11)	0.0263 (7)	0.000	0.000
C1	0.051 (3)	0.042 (2)	0.051 (3)	0.0113 (19)	0.000	0.000
Cl2	0.0977 (11)	0.0433 (7)	0.0849 (10)	−0.0220 (6)	0.000	0.000
C2	0.075 (3)	0.042 (2)	0.044 (2)	0.014 (2)	0.000	0.000
Cl3	0.0372 (6)	0.0463 (7)	0.0872 (10)	0.0073 (4)	0.000	0.000
C3	0.061 (3)	0.043 (2)	0.042 (2)	−0.009 (2)	0.000	0.000
Cl4	0.0572 (5)	0.0552 (6)	0.0734 (7)	0.0006 (3)	0.0064 (5)	0.0182 (4)
C4	0.039 (2)	0.034 (2)	0.046 (2)	0.0056 (17)	0.000	0.000
Cl5	0.0334 (6)	0.0666 (8)	0.1018 (12)	0.0007 (5)	0.000	0.000
C5	0.041 (2)	0.040 (2)	0.044 (2)	−0.0008 (18)	0.000	0.000
C6	0.036 (2)	0.039 (2)	0.061 (3)	0.0008 (17)	0.000	0.000

### Geometric parameters (Å, º)

**Table:**

N—C4	1.318 (6)	C2—C3	1.385 (8)
N—C3	1.321 (6)	Cl3—C4	1.739 (4)
Cl1—C2	1.724 (5)	Cl4—C6	1.774 (3)
C1—C2	1.381 (7)	C4—C5	1.400 (6)
C1—C5	1.381 (6)	Cl5—C6	1.783 (5)
C1—H1A	0.9300	C5—C6	1.519 (6)
Cl2—C3	1.719 (4)	C6—Cl4^i^	1.774 (3)
			
C4—N—C3	118.0 (4)	N—C4—Cl3	112.7 (3)
C2—C1—C5	120.5 (4)	C5—C4—Cl3	122.2 (3)
C2—C1—H1A	119.7	C1—C5—C4	115.4 (4)
C5—C1—H1A	119.7	C1—C5—C6	121.1 (4)
C1—C2—C3	118.4 (4)	C4—C5—C6	123.5 (4)
C1—C2—Cl1	119.6 (4)	C5—C6—Cl4	110.77 (18)
C3—C2—Cl1	122.0 (4)	C5—C6—Cl4^i^	110.77 (18)
N—C3—C2	122.6 (4)	Cl4—C6—Cl4^i^	109.2 (2)
N—C3—Cl2	116.0 (4)	C5—C6—Cl5	112.2 (3)
C2—C3—Cl2	121.4 (4)	Cl4—C6—Cl5	106.84 (17)
N—C4—C5	125.1 (4)	Cl4^i^—C6—Cl5	106.84 (17)
			
C5—C1—C2—C3	0.0	C2—C1—C5—C6	180.0
C5—C1—C2—Cl1	180.0	N—C4—C5—C1	0.0
C4—N—C3—C2	0.0	Cl3—C4—C5—C1	180.0
C4—N—C3—Cl2	180.0	N—C4—C5—C6	180.0
C1—C2—C3—N	0.0	Cl3—C4—C5—C6	0.0
Cl1—C2—C3—N	180.0	C1—C5—C6—Cl4	119.32 (19)
C1—C2—C3—Cl2	180.0	C4—C5—C6—Cl4	−60.68 (19)
Cl1—C2—C3—Cl2	0.0	C1—C5—C6—Cl4^i^	−119.32 (19)
C3—N—C4—C5	0.0	C4—C5—C6—Cl4^i^	60.68 (19)
C3—N—C4—Cl3	180.0	C1—C5—C6—Cl5	0.0
C2—C1—C5—C4	0.0	C4—C5—C6—Cl5	180.0

Symmetry code: (i) *x*, *y*, −*z*+1/2.

### Hydrogen-bond geometry (Å, º)

**Table:**

*D*—H···*A*	*D*—H	H···*A*	*D*···*A*	*D*—H···*A*
C1—H1*A*···Cl5	0.93	2.48	2.944 (5)	111
